# *Demodex* Species and Culturable Microorganism Co-Infestations in Patients with Blepharitis

**DOI:** 10.3390/life13091827

**Published:** 2023-08-29

**Authors:** Joanna Pyzia, Katarzyna Mańkowska, Maciej Czepita, Karolina Kot, Natalia Łanocha-Arendarczyk, Damian Czepita, Danuta I. Kosik-Bogacka

**Affiliations:** 1 Department of Ophthalmology, Independent Provincial Public Integrated Hospital “Arkońska”, Arkońska 4, 71-455 Szczecin, Poland; joanna11224@wp.pl; 2 Department of Microbiology, Immunology and Laboratory Medicine, Pomeranian Medical University, al. Powstańców Wielkpolskich 72, 70-111 Szczecin, Poland; katarzyna.mankowska@pum.edu.pl; 3 Department of Ophthalmology, Pomeranian Medical University, al. Powstańców Wielkpolskich 72, 70-111 Szczecin, Poland; 4 Department of Biology and Medical Parasitology, Pomeranian Medical University, al. Powstańców Wielkpolskich 72, 70-111 Szczecin, Polandnatalia.lanocha.arendarczyk@pum.edu.pl (N.Ł.-A.); 5 Independent Laboratory of Pharmaceutical Botany, Pomeranian Medical University, al. Powstańców Wielkpolskich 72, 70-111 Szczecin, Poland

**Keywords:** *Acinetobacter baumannii*, *Bacillus* spp., *Corynebacteriaceae*, *Demodex folliculorum*, *Staphylococcus aureus*, *Streptococcus pneumoniae*

## Abstract

We aimed to determine the prevalence of *Demodex* spp. and bacterial infection in patients with blepharitis and also to investigate the relationship between culturable microorganisms and *Demodex* spp. in this study. The study included patients diagnosed with blepharitis (*n* = 128) and volunteers without ocular problems (*n* = 113). Eyelash sampling was performed by epilating eight lashes, which were then tested for *Demodex* spp. using a light microscope. The examination consisted of assessing the patient’s vision with and without ocular correction and tonus in both eyes and a careful examination of the anterior segment of both eyes. Bacterial identification was performed based on morphological, physiological, and biochemical methods. The prevalence of *Demodex* spp. was 8.0% in patients from the control group and all patients with blepharitis. Isolated forms of *Demodex* spp. were detected in all infested patients in the control group and in 58% of patients with blepharitis. A total of 35% of patients with blepharitis had from three to nine forms of *Demodex* spp., and 7% of patients with blepharitis had more than 10 mites in every field of vision. We found a statistically significant relationship between *Demodex* spp. infestation and the occurrence of eye dryness and sensations of burning and tearing, redness of the conjunctiva, feeling of a foreign body, loss of eyelashes, *Meibomian gland* dysfunction, and cylindrical dandruff. There were statistically significant relationships between *Demodex* sp. infestation and the presence of hyperopia, Meibomian cysts, chronic eyelid inflammation, and the use of eyeglasses. There was also a statistically significant relationship between the occurrence of *Demodex* spp. and seborrheic dermatitis and diabetes mellitus. Culturable microorganisms of the *ocular surface* were found in 8.7% of participants who were uninfested and in all patients infested with *D. folliculorum*. We isolated *Staphylococcus aureus*, *Acinetobacter baumannii*, *Streptococcus pneumoniae*, *Klebsiella oxytoca*, and *Bacillus* spp. from the conjunctival sac only in patients infested with *D. folliculorum*. This indicates an increased probability of colonization by pathogenic bacteria in patients with demodicosis. Therefore, patients infested with *D. folliculorum* should undergo a microbiological examination of conjunctival swabs.

## 1. Introduction

Blepharitis is a chronic ocular inflammation of the eyelid margin [1,2]. It is most commonly associated with different ocular symptoms, including inflammation of the eyelid margins, a burning sensation, irritation, tearing, photophobia, blurred vision, and red eyes [3,4]. Blepharitis can present with a range of signs and symptoms and is associated with various dermatological conditions, including seborrheic dermatitis, rosacea, and eczema [5]. Its etiopathogenesis is not known exactly but is suspected to be multifactorial, including environmental factors, bacteria, viruses, and parasites [1,6].

*Demodex* mites of the phylum Arthropods are one of the most common permanent ectoparasites in humans [7]. Two *Demodex* species can be found in humans: *D. folliculorum* (Simon, 1842) and *D. brevis* (Akbulatova, 1963) [8]. An adult *D. folliculorum* reaches a length of 0.3–0.4 mm and occurs in the hair follicles and Zeiss glands, usually forming clusters of several individuals [9]. *Demodex brevis* is similar to *D. folliculorum* but shorter, reaching a length of 0.2–0.3 mm [8]. It is most often isolated as separate specimens in the depths of sebaceous glands in the skin of the face, in the Meibomian glands, and in the eyelids [8]. The life cycle of *Demodex* spp. lasts between 14 and 18 days (approximately 14.5 days), during which the mites thrive in only one host [9,10]. *Demodex* spp. have five stages: egg, larva, protonymph, nymph, and adult forms [11,12]. All stages of *Demodex* spp. exhibit much vitality, especially in a moist and dark environment [11].

The rate of *Demodex* infestation increases with age and has been noted in 100% in those over 70 years [13]. The transmission routes of *Demodex* spp. have not yet been fully investigated. It is likely that infection with *Demodex* spp. occurs through direct contact, the use of common toiletries or towels, or by airborne eggs and dust [10,11]. Skin colonization occurs during childhood or adolescence; no mites are found in the skin of newborns [14,15].

It has been reported that 42–81% of blepharitis patients have concomitant infestations with *Demodex* mites [16,17,18]. Studies support correlations between ocular demodicosis and anterior blepharitis, Meibomian gland dysfunction (MGD), chalazia, and keratoconjunctivitis [19]. Therefore, *D. folliculorum* and *D. brevis* are recognized to cause blepharitis [3,20]. *D. folliculorum* is thought to cause chronic anterior blepharitis, while *D. brevis* is considered to cause posterior blepharitis [13].

The role of *Demodex* spp. in the pathogenesis of blepharitis is unclear, but it likely involves direct damage from *Demodex* mites and their metabolites, including mechanical abrasions caused by the mite’s claws, Meibomian gland orifice blockage, and consumption of epithelial cells, carrying bacteria [21]. Through their digestive tract, microbes are transported to the hair follicles of the host [17,22]. The disintegration of *Demodex* spp. inside the hair follicle can lead to the release of the transmitted bacteria and the development of a local immune response [23]. *Demodex* spp. are the only organisms to create cylindrical dandruff, and the deposits may include lipids, keratin, *Demodex* eggs, and dead *Demodex* mites [24].

*Demodex* is mainly thought to be a vector for bacteria such as *Streptococcus* spp., *Staphylococcus* spp., *Propionibacterium acnes*, *Corynebacterium* spp. or *Bacillus oleronius*, as well as for viruses and fungi [18,25,26,27]. The bacteria colonizing conjunctival sacs are *Staphylococcus* spp., *Streptococcus* spp., *Micrococcus* spp., and *Corynebacterium* spp., occurring in about 70%, 26%, 22%, and 7% of patients, respectively. The most common among obligate anaerobic bacteria are *Propionibacterium acnes* (~44%), *Peptostreptococcus* spp. (~6%), *Lactobacillus* spp. (~2%), and *Clostridium* spp. (1%) [28].

The composition of the culturable microorganisms of the conjunctival sac depends on many factors, including patient age and the presence of chronic disease. For example, *Propionibacterium* spp. are more common in adults, whereas *Streptococcus* spp. are more common in children [29]. Chronic diseases, including diabetes mellitus, may increase the number of coagulase-negative staphylococci compared to healthy patients. These bacteria produce substances inhibiting the development of pathogenic bacteria, stimulating local immunological processes, and the exfoliation and regeneration of epithelial cells of the eye [30].

In this study, we aimed to determine the prevalence of *Demodex* spp. and bacterial co-infections in patients with blepharitis and also to investigate the relationships between culturable microorganisms and *Demodex* spp. in this study. The identification of microbial communities on the ocular surface of *Demodex* blepharitis patients can help clarify the pathological mechanisms and provide valuable information for prevention or treatment.

## 2. Materials and Methods

A *randomized* double-blind placebo-controlled study was carried out between October 2015 and May 2018 and was approved by the Bioethics Committee of the Pomeranian Medical University in Szczecin (KB-0012/82/15). It conformed to the principles outlined in The Declaration of Helsinki as revised in 2008. The patients who participated in the study were informed about the study’s process and signed an agreement beforehand. At any stage of this project, the participants had the option to withdraw from it. All samples collected from the patients were labeled with a secret code number, which was confidential and closely protected from third parties.

### 2.1. Characteristics of Groups

Patients from north-western Poland were divided into two groups: participants without ocular problems (*n* = 113, group I) and patients diagnosed with blepharitis (*n* = 128, group II). Group two was further divided into two groups: patients from hospitals and outpatient settings (*n* = 100, subgroup IIA) and residents of the Social Welfare Home (SWH) in Jaromin (*n* = 28, subgroup IIB). The residents of the SWH in Jaromin were individuals with psychotic disorders (e.g., schizophrenia, schizoaffective disorders, and delusional disorders) and intellectual disabilities.

Group I included females (*n* = 77) and males (*n* = 36) aged 3 to 81, with a mean age of 52.0. Control participants in this group had never been diagnosed with blepharitis, and they had clean eyelashes without cylindrical dandruff or squamous cell debris (collarette).

Patients from group IIA (60 females and 40 males aged 23 to 90; mean age 62.9). They were selected from the Ophthalmology Department of the Regional Hospital in Kołobrzeg and the Ophthalmology Department of the Independent Public Complex of Health Care Centres in Gryfice. The residents of SWH in subgroup IIB were 28 males aged 41 to 80 (mean age 59.0).

The diagnosis of blepharitis was made by demonstrating any two of the following symptoms: (1) burning sensation in the eyes, (2) tearing, (3) eyelid hyperemia, (4) foreign body sensation, and (5) excessive loss of eyelashes. The exclusion criteria for both groups of patients were as follows: using topical ophthalmic medications (except artificial tears) in the 3 months before the study started, a history of ocular or eyelid trauma and surgery in the last 6 months, previous diagnosis of chemical burns, Stevens–Johnson syndrome, ocular cicatricial pemphigoid, with eyelid malpositions such as entropion, ectropion, and distichiasis, signs of active ocular infestation, or inflammation other than blepharitis.

Participants were also excluded if they had used any systemic or topical antibacterial/antiparasitic/steroidal drug, topical tea tree oil (TTO), hypochlorous acid, or any other lid hygiene products (lid scrubs) within the last 14 days. Participants from both groups were also excluded if they had used contact lenses, artificial eyelashes, or eyelash extensions within the last 14 days. Participants with lid structural abnormalities, surgery of the eyelid margin, acute ocular infection, or inflammation other than blepharitis, dry eye, and hypersensitivity to lotilaner were also excluded.

All participants from both groups were asked to provide informed consent to participate in the study, followed by an interrogation to collect information and slit-lamp evaluation with a magnification of ×25. Additionally, all patients were interviewed using a questionnaire to collect data on demography, health status, and chronic diseases.

Patients from the two groups underwent parasitological and microbiological examinations.

### 2.2. Demodex *spp.* Examination

A total of eight eyelashes were excised per patient in both groups, with four lashes taken from each eye using sterile disposable plastic tweezers. This was performed according to standard parasitological methods applied in cases of suspected *D. folliculorum* or *D. brevis* infestations. The extracted eyelashes were placed separately on each end of a slide and coated with Hoyer medium before a coverslip was placed on top.

The presence and counting of *Demodex* were performed in the samples using light microscopy with magnifications of 4×, 10×, and 40×. Infestation was defined as the presence of eggs, larvae, or mature forms of *Demodex* spp. on the eyelashes.

The intensity of *Demodex* spp. infestation using a 40× magnification was categorized using four levels of parasite load: (+) low—single (≤ two mites) mites in almost every field of vision; (++) medium—3–9 mites in every area of vision; and (+++) high—>10 mites in every field of vision. The results were archived using a camera.

### 2.3. Treatment of Demodicosis

Patients who showed evidence of *Demodex* spp. infestation in microscopic examinations were treated according to the following protocol, which spanned a minimum of 9 weeks. They were advised to apply an external ointment composed of metronidazole (0.5 g), glycerin (2.0 g), and vaseline (20.0 g) using a disposable applicator on the eyelids, eyebrows, and sides of the nose 2 times a day (in the morning and evening). Additionally, maintaining proper hygiene of the eyelid edges was recommended to alleviate cylindrical dandruff. Based on our observations, the procedure of daily hygiene of the eyelid margins was developed. Each patient began their daily morning routine by gently applying a diluted children’s shampoo onto the eyelids of both eyes with a gauze pad, allowing it to sit for a few minutes, and then rinsing off the residual foam with warm water. Four to six times a day, patients were instructed to cleanse the eyelid edges, eyebrows, and facial skin with Demoxoft solution multiple times. This solution contained aloe extract and Oliv 300^®^, known for their ability to reduce dryness and damage to the skin, along with Fucocert^®^, D-panthenol, and hyaluronic acid, which provided hydration to the eyelid skin and effectively stimulated the regenerative processes.

After the 9-week period, patients were required to return for a follow-up visit to assess their progress. If *Demodex* spp. were found on the eyelashes, symptoms persisted (redness of eyelid margins and a foreign body sensation), and vision deteriorated, the treatment regimen was repeated. Some patients chose to have follow-up visits at 18 and 27 weeks into the treatment. If, after the 9-week treatment period, the presence of *Demodex* spp. was not detected in patients, ocular symptoms were not observed, and vision remained the same or improved, the treatment was considered concluded. Additionally, patients diagnosed with bacterial infections received supplemental antibiotic therapy.

### 2.4. Clinical Examination

Participants took part in an ophthalmic interview to gather information about any eye problems they experienced, as well as their personal and familial history of eye diseases. However, the residents from the Social Welfare Centre were not extensively examined due to their limited cooperation during the examination.

The ophthalmological examination involved testing the uncorrected and best-corrected distance visual acuity (VA) using Snellen charts. The examination was conducted in a room with consistent lighting conditions, and the testing distance was set at 4 m. The results of the best-corrected visual acuity were recorded and converted to the LogMAR scale (decimal logarithm of the minimum angle of resolution) for analysis.

Intraocular pressure (IOP) was measured using a Mackay-Marg Tono-Pen AVIA applanation tonometer (Ametek Reichert Technologies, Depew, NY, USA). The measurement was taken three times, and the average value was used for analysis.

Furthermore, an anterior segment examination was performed using a Haag-Streit L0185 slit lamp (Nikon Corporation, Tokyo, Japan).

### 2.5. Microbiological Examination

The samples for microbiological examination were obtained from the conjunctival sac using a sterile swab and AMIES transport medium. The samples were promptly delivered to the microbiological laboratory for analysis. The identification methods used in this paper were consistent with those commonly employed in routine bacteriological diagnostics.

The samples were plated on basic microbiological media, including Columbia agar with 5% sheep blood, Chapman, MacConkey, chromogenic media, and Sabouraud. These plates were then incubated at 37 °C for 24–48 h. The identification of strains was based on the morphological evaluation of colonies on the media and preparations stained by the Gram method.

For the identification of *Staphylococcus* spp., the morphology in Gram staining, hemolytic capacity of colonies on Columbia agar medium with 5% sheep’s blood, and growth evaluation on Chapman medium and Chromid^®^ *S. aureus* Elite were utilized. This allowed for the differentiation of staphylococci into mannitol-positive and mannitol-negative strains. Strains that could ferment mannitol were further analyzed for the presence of clumping factor A, protein A using the Staphylotect Plus Latex Agglutination Test (Thermo Scientific, Waltham, MA, USA), and tube coagulase. The presence of all three factors indicated the presence of *Staphylococcus aureus*. All strains that grew as pink colonies on Chromid^®^ *S. aureus* Elite were classified as *Staphylococcus* aureus species.

For the identification of *Streptococcus pneumoniae*, the morphology in Gram staining and the hemolytic capacity of colonies on Columbia agar medium with 5% sheep’s blood in the presence of an optochin disc were used. All strains with α-hemolysis and resistance to optochin were classified as *Streptococcus* pneumoniae.

All bacteria growing in the form of grey, large colonies on Columbia agar medium with 5% sheep’s blood were identified as bacteria of the genus *Bacillus*. Gram staining (Gram-positive bacilli with spores) and VITEK Compact (bioMerieux, Warsaw, Poland) identification allowed for the classification of the bacteria as *Bacillus* subtilis species.

MacConkey medium and Chromid^®^ CPS^®^ Elite were used to isolate and identify strains of Gram-negative rods. As this group of microorganisms is not usually pathogenic in conjunctivitis, only growth morphology on the medium was evaluated, dividing bacteria into lactose-positive and lactose-negative strains. On chromogenic media, bacteria grew as transparent (*Acinetobacter baumannii*) and green (*Klebsiella oxytoca*) colonies. The species identification was performed using VITEK Compact (bioMerieux, Poland).

All microorganisms showing growth characteristics of *Corynebacteria* on Columbia agar with 5% sheep blood were analyzed by Gram staining. Gram-positive rods with a characteristic club-like shape were considered to be *Corynebacterium* spp.

The drug susceptibility of isolated strains was determined using the disk diffusion test. The antibiogram was performed for *Staphylococcus aureus* strains, as this pathogen is known to cause conjunctivitis. A suspension of density 0.5 according to McFarland scale (1 × 10^8^ CFU/mL) was prepared from single colonies grown after 18–24 h. This suspension was then inoculated into Mueller–Hinton agar medium (bioMerieux, Poland). Antibiotic discs containing erythromycin (15 µL), clindamycin (2 µL), gentamicin (10 µL), neomycin (10 µL), tetracycline (10 µL), and trimethoprim/sulfamethoxazole (1.25/23.75 µL) were placed onto the culture medium. The determination of methicillin-resistant *Staphylococcus aureus* (MRSA) was performed using cefoxitin 30 μg disks. The growth inhibition zone around the discs was assessed, and the results were analyzed according to the guidelines provided by the National Reference Centre for Microbial Susceptibility (www.eucast.org). The bacterial susceptibilities were recorded as “resistant”, “intermediate”, and “sensitive”.

**Figure 1:**
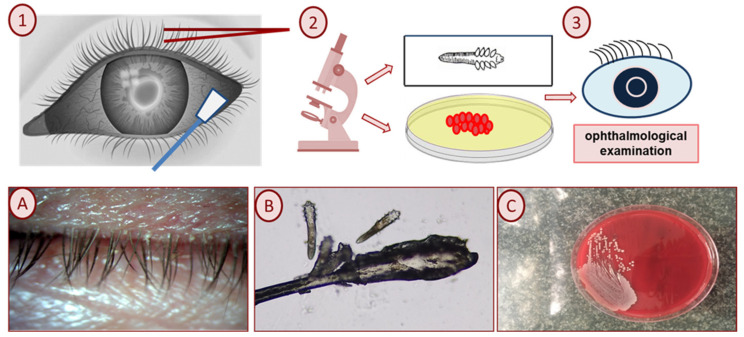
Schematic illustration of research methodology. (**1**,**2**) Parasitological examination of eyelashes and microbiological examination from the conjunctival sac. (**3**) Clinical examination; (**A**,**B**) collarettes, the pathognomonic sign of *Demodex* blepharitis: lash sampling and microscopic examination reveal cylindrical dandruff harboring mites; and (**C**) *Staphylococcus aureus* on Columbia agar with 5% defibrinated sheep blood.

The scheme of the study is presented in Figure 1.

### 2.6. Statistical Analysis

Statistical studies were performed using Stat Soft Statistica 10.0 PL. Participants infested with *Demodex* spp. were excluded from the comparisons between the control group and other groups. The assumption of normal distribution for quantitative variables (tonus and visus) was checked using the Shapiro–Wilk test. The nonparametric Mann–Whitney test was used for the comparisons of intraocular pressure (IOP) and visual acuity (VA) between uninfested and *Demodex folliculorum*-infested patients. To explore possible relationships between *D. folliculorum* infestation and the occurrence of eye diseases and symptoms in patients from the two groups, the chi-square independence test was used. Differences were deemed statistically significant at *p* < 0.05.

## 3. Results

### 3.1. Prevalence of Demodex *spp.* Infestation

*Demodex folliculorum* infestation was observed in nine participants (8.0%), including three women (3.8%) and six men (17.6%) in group I (control group). The presence of single (≤two mites) adult forms of *Demodex* spp. in almost every field of vision was found in patients aged 18–59 (6.1%), 60–69 (10%), and 70–74 (20%).

In patients with blepharitis (group II), *Demodex* spp. were observed in all patients. *Demodex folliculorum* was reported in all participants, but one man also had *D. brevis*. Among patients with blepharitis, 58% had ≤2 mites observed in almost every field of vision, 35% had 3–9 mites in every area of vision, and 7% had >10 mites in every field of vision (Table 1). The intensity of *Demodex* spp. infestation was similar in women and men.

In subgroup IIA, among 20 females, only adult forms of *Demodex* spp. were found; in 30 females, both adult forms and nymphs were found; and in 10 females, all developmental forms of *Demodex* spp. were observed. Among all men with blepharitis, adult forms of *D. folliculorum* were detected. A total of 20 male patients had larvae forms, and 11 male participants also had eggs of *Demodex* spp. In subgroup IIB (residents of the SWH), 24 participants had single forms of *D. folliculorum*, 3 had many mites, and 1 participant had a very numerous mite presence (Table 1). In all residents of the SWH, adult forms were detected, but six participants also had larvae and nymphs, and one subject also had eggs of *Demodex* spp.

### 3.2. Demodex *spp.* Prevalence and Chronic Disease

The relationships between the *Demodex* spp. infestation and the occurrence of comorbidities, including seborrheic dermatitis (SD), diabetes mellitus (DM), rheumatoid arthritis (RA), and cancers were analyzed. Seborrheic dermatitis was reported in three (3) residents of the SWH (10.7%). Diabetes mellitus occurred in two (2) participants in the control group, nineteen (19) patients in group IIA, and one (1) in group IIB. *Demodex* spp. infestation was reported in all DM patients with blepharitis, while patients in the control group did not show *Demodex* spp. infestation. Infestation with *Demodex* spp. was observed in three (3) patients with RA, all of whom had blepharitis. Additionally, infestation with *Demodex* spp. was diagnosed in five patients with cancers. There was a statistically significant difference between the *Demodex* spp. infestation and SD (Chi2 = 16.6; DF = 2; *p* < 0.001) and DM (Chi2 = 9.8; DF = 2; *p* = 0.007). There were no statistically significant relationships between the occurrence of parasites and RA and cancers.

### 3.3. Demodex *spp.* Prevalence and Ocular Symptoms

Table 2 presents the frequency of ocular symptoms occurring in patients uninfested and infested with *Demodex* spp. The symptoms were selected based on our observations and data from the literature. Patients infested with *Demodex* spp. from group I were excluded from this analysis. The results showed statistically significant relationships between *Demodex* spp. infestation and the occurrence of dry and burning sensations in the eyes, lachrymation, conjunctival redness, foreign body sensation, and lash loss.

The comparison between uninfested (control group) and *Demodex folliculorum*-infested patients shows that the mean IOP and VA were significantly lower in infested participants than in uninfested participants (Table 3).

*Meibomian gland* dysfunction and cylindrical dandruff were observed in 28 (21.9%) controls and 39 (30.5%) patients infested with *D. folliculorum*. There was a statistically proven relationship between the occurrence of *D. folliculorum* and *Meibomian gland* dysfunction (*p* < 0.000) as well as cylindrical dandruff (*p* < 0.000).

Most subjects (*n* = 121; 52.2%) *wore glasses*, including 35 (33.7%) uninfested and 86 (67.2%) infested with *D. folliculorum*. A statistically significant relationship was observed between *D. folliculorum* infestation and *wearing glasses* (*p* < 0.001).

### 3.4. Treatment of Demodicosis

The course of treatment was analyzed in 33 individuals infected with *Demodex* spp. with symptoms of blepharitis. Microscopic examination of eyelashes before treatment showed a predominance of larval and mature forms of *Demodex* spp. Patients complained of burning and tearing and, less frequently, of a foreign body sensation. Slit-lamp examination revealed abnormal growth of eyelashes, cylindrical dandruff, and Meibomian gland blockage. After 9 weeks of treatment, 10 patients (four women and six men) showed no developmental forms of *Demodex* spp. These individuals had small cylindrical dandruff and slight Meibomian gland blockage.

In the remaining patients (*n* = 23), microscopic examination showed the presence of individual mature forms, and the slit lamp examination revealed smaller cylindrical dandruff and reduced Meibomian gland blockage. After treatment, these patients reported either no discomfort or only slight discomfort due to the foreign body sensation. Visual acuity remained unchanged in most patients after treatment, while in four patients, it improved by one to two lines on the Snellen chart.

At the second follow-up after 18 weeks of treatment, nine patients returned for examination. Microscopic examination of their eyelashes revealed individual mature forms of *Demodex* spp. The slit lamp examination showed smaller cylindrical dandruff and slightly paler eyelid margins. Visual acuity improved by one additional line on the Snellen chart in seven patients, and intraocular pressure was 2 mmHg lower than before treatment in seven patients.

Only five patients returned for the third follow-up. Microscopic examination revealed no developmental forms of *Demodex* spp. in four patients. However, the 80-year-old patient still had larval and mature forms of *Demodex*. Slit-lamp examination of the 80-year-old patient showed a reduction in telangiectasia on the upper eyelid and less redness of the eyelid margins, as well as a decrease in cylindrical dandruff. In the remaining three patients, symptoms decreased, and one female patient showed no initial symptoms. Slit-lamp examination indicated reduced cylindrical dandruff and less redness of the eyelid margins. Visual acuity improved by two lines on the Snellen chart in three female patients, and in all patients, intraocular pressure decreased by 2–3 mmHg.

### 3.5. Demodex *spp.* and Microorganisms

The normal *ocular surface* culturable microorganisms were found in nine (8.7%) uninfested participants and all patients infested with *D. folliculorum*. The aerobic and facultative anaerobic culturable microorganisms colonizing the conjunctival sacs of the examined patients were *Bacillus subtilis*, *Corynebacterium* spp., *Haemophilus influenzae*, *Micrococcus* spp., *Staphylococcus* spp., and *Streptococcus* spp. Four (3.1%) patients infested with *D. folliculorum* had *Corynebacteriaceae*, three (2.3%) of whom also had chalazia.

Only in patients infested with *D. folliculorum* did we isolate *Staphylococcus aureus* (*n* = 9, 7%), *Acinetobacter baumannii* (*n* = 1, 0.8%), *Streptococcus pneumoniae* (*n* = 1, 0.8%), *Klebsiella oxytoca* (*n* = 1, 0.8%), and *Bacillus* spp. (*n* = 1, 0.8%) in the conjunctival sac.

### 3.6. Case Reports

**Figure 2:**
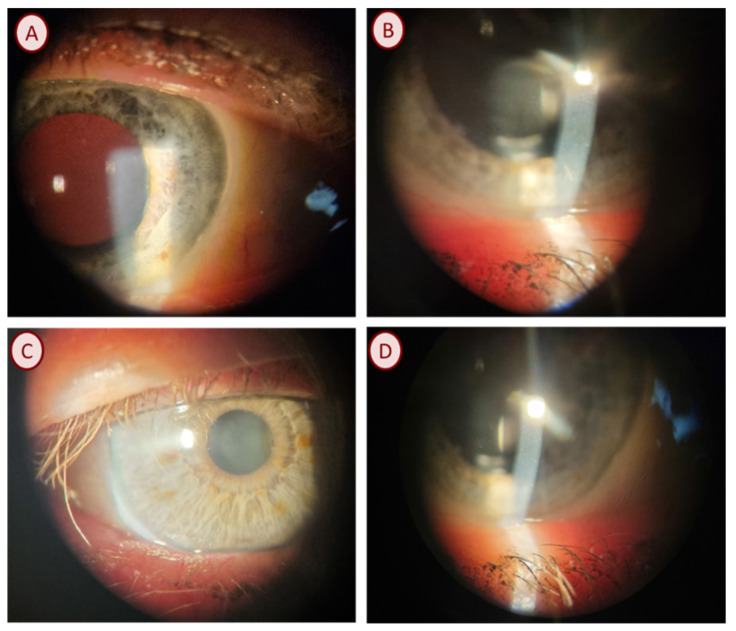
Clinical manifestation of *Demodex* blepharitis. (**A**). Meibomian gland dysfunction, keratin dandruff, mild blepharitis. (**B**). Meibomian gland dysfunction (visible tear film), eyelid margin telangiectasia, standing tear symptom (watery eye). (**C**). Meibomian gland obstruction, eyelid margin telangiectasia. (**D**). Meibomian gland obstruction.

*Staphylococcus aureus* was observed in an adult male patient with no chronic diseases. Mature forms of *D. folliculorum* were observed on the eyelashes in the microscopic examination. Ophthalmic examination showed that the visual acuity (VA) of the right and left eye was 1.0, and the intraocular pressure (IOP) of the right eye was 11.7 mmHg and 10.7 mmHg, respectively. Anterior segment examination using a slit-lamp showed cylindrical dandruff on the upper eyelid and blockage of the Meibomian glands (Figure 2A).

*Staphylococcus aureus* was also found in an adult female patient with arterial hypertension and diabetes mellitus. Microscopic examination of her eyelashes revealed numerous larval and mature forms of D. folliculorum. Ophthalmic examination showed that the VA of the right eye was 0.9 and that of the left eye was 1.0. The intraocular pressure was 12.0 mmHg and 13.0 mmHg, respectively. The patient suffered from irritation of the eye and conjunctiva.

*Staphylococcus aureus* sensitive to erythromycin, clindamycin, gentamicin, neomycin, tetracycline, and trimethoprim/sulfamethoxazole were observed in two patients with mature forms of *D. folliculorum*. An adult female patient with thrombocytopenia without ophthalmic symptoms had hyperopia corrected by glasses. Ophthalmic examination showed that the VA was 0.2 in both eyes and the IOP was 14.0 and 15.0 mmHg, respectively. The slit-lamp examination showed slight follicular irritation of the conjunctiva.

In an adult female patient with hyperopia corrected with glasses, VA was 0.6 in the right eye and 0.8 in the left eye. The intraocular pressure was 17.0 mmHg in both eyes. Slit-lamp examination showed cylindrical dandruff on the upper eyelid. *Staphylococcus aureus* was found. Microscopic examination of his eyelashes revealed mature forms of *D. folliculorum*. Due to poor cooperation, the visual acuity of the eyes was not examined. The intraocular pressure was 14.0 in the right eye and 15.0 mmHg in the left eye. Examination with a slit lamp showed a single instance of cylindrical dandruff on the upper eyelid.

*Staphylococcus aureus* was found in the patient from the SWH. Microscopic examination revealed isolated mature forms of *D. folliculorum*. Similarly, visual acuity was not examined due to lack of cooperation; the IOP was 15.0 mmHg in both eyes. The slit-lamp examination showed irritation of the conjunctiva close to the upper and lower eyelids, and both the upper and lower eyelids were swollen. Additionally, *S. aureus* was found. Isolated mature forms of *D. folliculorum* were found in the microscopic examination around the patient’s eyelashes. Visual acuity was 1.0 in both eyes, while the IOP was 9.0 in the right eye and 11.0 mmHg in the left eye. Examination using a slit lamp showed no specific symptoms.

Methicillin-resistant *S. aureus* was found in an adult male patient with hyperopia corrected by glasses, hypertension, and atrial fibrillation. Numerous mature forms of *D. folliculorum* were observed in the patient. Ophthalmic examination showed that the VA in both eyes was 1.0, and the IOP was 21.0 in the right eye and 18.0 mmHg in the left eye. On the upper eyelid, we observed cylindrical dandruff and blockage of the Meibomian glands; eyelashes were glued together. MRSA was also found in an adult HIV-infected patient from the SWH. Microscopic examination showed isolated mature forms of *D. folliculorum*. The intraocular pressure was 11.0 mmHg in both eyes. Slit-lamp examination showed pale conjunctiva.

*Acinetobacter baumannii* was isolated from an adult patient with hypertension. The patient had numerous eggs (Figure 1B) and the larval and mature forms of *D. folliculorum*. The best-corrected distance visual acuity was found to be 0.7 in the right eye and 0.6 in the left eye. The intraocular pressure was 20 mmHg in the right eye and 17 mmHg in the left eye, respectively. The slit-lamp test revealed cylindrical dandruff on the upper eyelid.

*Streptococcus pneumoniae* was found in an adult patient with mature forms of *D. folliculorum.* Due to poor cooperation, the visual acuity of the eyes was not examined. The intraocular pressure was 12.0 in the right eye and 9.0 mmHg in the left eye. The slit-lamp study showed irritation, conjunctival hyperemia, cylindrical dandruff, and Meibomian gland dysfunction (Figure 2B).

*Klebsiella oxytoca* was found in an adult patient with hypertension. During the microscopic examination of eyelashes, the patient had numerous eggs and the larval and mature forms of *D. folliculorum*. Ophthalmological examination showed that the VA was 0.3 in the right eye and 0.4 in the left eye. The intraocular pressure was 21.0 mmHg in the right eye and 18.0 mmHg in the left eye. In addition, the patient had hyperopia corrected with glasses. In the ophthalmological examination, cylindrical dandruff was observed, the eyelashes were stuck together, and the Meibomian glands were blocked with an oily secretion (Figure 2C,D).

*Bacillus* spp. were found in an adult man without chronic diseases. The patient presented with isolated mature forms of *D. folliculorum* during microscopic examination. Due to poor cooperation from the patient, the vision was not examined, whereas the IOP was 14.0 mmHg in the right eye and 21.0 mmHg in the left eye. The slit-lamp examination did not show any changes.

## 4. Discussion

Blepharitis is one of the most common ocular disorders in daily ophthalmological practice [3]. Several plausible mechanisms by which *Demodex* spp. contribute to blepharitis include direct damage, acting as a vector for bacteria, and inducing hypersensitivity and inflammation [31]. Although this mite has been found in patients with chronic blepharitis, it has also been found on the eyelids of patients without blepharitis. The presence of ocular *Demodex* spp. colonization is associated with the occurrence of eye dryness, sensations of burning and tearing, redness of the conjunctiva, feeling of a foreign body, loss of eyelashes, Meibomian gland dysfunction, and cylindrical dandruff.

In the present study, treatment using an ointment containing metronidazole, as well as washing the eyelid and eyebrow margins with Demoxoft fluid and diluted children’s shampoo in the control group, resulted in complete eradication of mites after 9 weeks of therapy. This could be due to the low intensity of infestation and the small group of patients (*n* = 3). In patients with symptoms of eyelid inflammation, the same treatment scheme resulted in a reduction in the intensity of infestation, a decrease or resolution of symptoms, and improvement in ophthalmological results, especially after 27 weeks of treatment.

Wearing glasses has been linked to *Demodex* infestation in patients with seborrheic dermatitis and diabetes mellitus. Severe cases of blepharitis can arise from co-infestation of *Demodex* spp. and bacteria, with the high prevalence of *Demodex* spp. being accompanied by a higher abundance of certain bacteria on the ocular surface [32].

Some authors suggest that *Demodex* infestation may reduce the diversity of the microbiome in the conjunctival sac, thereby destabilizing it [31]. In this study, culturable bacteria were found in conjunctival sac swabs in all patients infested with *Demodex* spp. and in about 9% of uninfested participants. This may indicate that *Demodex* spp. promote colonization of the conjunctival sac with culturable microbiota.

Using bacterial culture methods, Zhu et al. [21] found bacteria in 54 patients with blepharitis (45 of them were also infested with *Demodex* spp.) and 37 without blepharitis. The colony counts and the incidence of *Propionibacterium acnes* from *Demodex* spp.-infested patients were significantly higher than in non-infested patients. Lee et al. [33] reported an increase in *Staphylococcus* spp., *Corynebacterium* spp., and *Enhydrobacter* spp. and a decrease in *Propionibacterium* spp. in patients with blepharitis (*n* = 7). Yan et al. [32], using 16S rRNA gene sequencing, demonstrated that *Firmicutes*, *Proteobacteria*, *Actinobacteria*, *Bacteroidetes*, and *Cyanobacteria* spp. were the main culturable microorganisms in patients with (*n* = 30) and without (*n* = 14) *Demodex* spp. However, the studies were conducted in a small number of patients around 41 years of age. In our study, we also detected members of other skin taxa in patients with *Demodex* infestation, such as *Lactobacillus*, *Bacteroides*, *Bifidobacterium*, *Micrococcus*, and *Acinetobacter*, at a relative abundance of 1% in more than half of the samples.

Spickett [34] showed that *D. folliculorum* might be a vector for *Mycobacterium leprae*. *Demodex* mites may also transmit *Staphylococcus* spp. and *Streptococcus* spp. on their surface. In a study conducted on patients, staff, and visitors of the Optometry Clinic in Oklahoma, *S. aureus* and *S. epidermidis* were found in ~20% and ~80% of participants, respectively [22]. The study reported that two or more mites (about 10% and 5%, respectively) were found more frequently in patients infested with S. aureus than in uninfested patients. *Staphylococcus aureus* was found in ~20% of patients aged 1–29 years, in about 10% of patients aged 30–59, and in ~15% of patients aged 60–89. In another study, Türk et al. [35] found *S. aureus* in two *D. folliculorum*-infested patients with blepharitis. In our study, *S. aureus* was isolated from 10% of *D. folliculorum*-infested patients, including about 15% of the Social Welfare House residents. One nursing home resident with psychotic disorder had co-infestation with *Demodex* spp. and methicillin-resistant *Staphylococcus aureus*. We did not find *S. aureus* in the uninfested participants.

Lee et al. [36] found no differences in the presence or distribution of bacteria on eyelashes between uninfested and *Demodex* spp.-infested patients. Coagulase-negative *Staphylococcus* spp., *Corynebacterium diphtheriae*, and *S. aureus* were found in patients of both groups. There were no differences in MRSA occurrence on eyelids between uninfested and *Demodex* spp.-infested patients. Zhu et al. [21] did not observe differences in colonies of *S. aureus* and *S. epidermidis* between *Demodex* spp.-infested and uninfested patients. Bezza Benkaouha et al. [37] also did not find a difference in culturable microorganisms and *Demodex* spp. infestation; however, the authors conducted a study on a small number of subjects.

*Acinetobacter baumannii* is one of the most common etiological factors of hospital-acquired infections. It shows natural mechanisms of resistance to antibiotics and chemotherapy. In the present study, *A. baumannii* was isolated from the conjunctival sac of a patient infested with *D*. *folliculorum.*

Lacey et al. [38] isolated *Bacillus oleronius* from a *D. folliculorum* extracted from the face of patients with papulopustular rosacea and stated that two specific antigens (62 and 83 kDa) produced by this bacteria can stimulate and be responsible for inflammation of the hair follicle. Li et al. [39], on serum from 59 patients with diagnosed rosacea, showed a statistically significant correlation between ocular *Demodex* infestation and serum immunoreactivity and 62 and 83 kDa *B. oleronius* proteins.

O’Reilly et al. [40] showed that proteins derived from *B. oleronius* might be a neutrophil-activating factor. Such neutrophil activation could occur if *B. oleronius* proteins released from mites entered the tissues surrounding the hair follicle. This, in turn, could result in the development of local inflammation in the perifollicular tissue. In our study, *Bacillus* spp. were isolated from the conjunctival sac of a patient with *D. folliculorum* infestation.

Szkaradkiewicz et al. [41] isolated 23 strains of *Bacillus oleronius* from 18 patients with *Demodex*-related chronic blepharitis. The authors observed more severe symptoms of blepharitis in patients with *B. oleronius* infestation. However, *B. oleronius* was also found in five uninfested participants, which may undermine its role in developing blepharitis. The authors concluded that these bacteria, living inside the intestines of the *Demodex* mites as symbionts, can be excreted by these mites onto the surface of human skin. Due to the fact that *B. oleronius* plays a significant role in the process of digestion in termites, it seems that these bacteria may play a similar role in *Demodex* spp. [35,41].

*Streptococcus pneumoniae* can cause inflammation of the middle ear, paranasal sinuses, conjunctiva, and cornea of the eye, as well as pneumonia. *Streptococcus pneumoniae* infection can cause severe or chronic complications [42,43]. In the present study, *S. pneumoniae* was reported in a resident of a Social Welfare Home infested with *D*. *folliculorum.*

The present study was a preliminary study that demonstrated the concurrence of some bacteria and *Demodex* spp., but it has some limitations that should be addressed. We recruited only healthy patients (non-infested and without blepharitis) and patients with blepharitis who were also infested with *Demodex* spp. As blepharitis can be caused not only by *Demodex* spp. but also by various bacterial infections, future research should also include patients with blepharitis without *Demodex* spp. infestation. In addition, in our study, we did not distinguish between mixed and single types (e.g., anterior or posterior) of blepharitis, although Rynerson and Perry [44] observed disruption of the biofilm in the eyelids in different types of blepharitis. Finally, future research should involve larger groups of patients, which would allow analyses of the subtypes of bacteria and *Demodex* spp.

## 5. Conclusions

*Demodex* spp. can collect microorganisms found on the surface of the skin and transport them to the host’s hair follicles. Transmission of bacteria from non-susceptible sites to sensitive areas can contribute to the development of inflammatory reactions. Therefore, patients infested with *Demodex* spp. should also undergo a microbiological examination of conjunctival swabs. The treatment of each patient should be individualized and adapted to the clinical condition, and in cases of bacterial co-infection, an antibiotic and/or a topical steroid drug should be additionally prescribed. Finally, daily hygiene of the eyelid margins should be recommended.

## Figures and Tables

**Table 1:** The intensity of *Demodex folliculorum* and *D. brevis* in patients from north-west Poland (+, single (≤two mites) mites in almost every field of vision; ++, 3–9 mites in every field of vision; and +++, >10 mites in every field of vision).

Group	Sex	Intensity of *Demodex* spp. (n, %)		
		+ n/%	++ n/%	+++ n/%
I	female	3/100	-	-
	male	6/100	-	-
	Total	9/100	-	-
II	female	36/60.0	19/31.7	5/8.3
	male	46/67.7	19/27.9	3/4.4
	Total	82/64.1	38/29.7	8/6.2
IIA	female	36/60.0	19/31.7	5/8.3
	male	22/55.0	16/40.0	2/5.0
	Total	58/58.0	35/35.0	7/7.0
IIB	male	24/85.7	3/10.7	1/3.6
	Total	24/85.7	3/10.7	1/3.6

**Table 2:** The frequency of ocular symptoms occurring in non-infested patients (group I) and those infested with *Demodex* spp. (subgroups IIA over IIB) (BP, blepharitis; Chi2; Test χ^2^; and df, number of degrees of freedom).

Symptom	Patients							
	Group I		Subgroup IIA		Subgroup IIB		Total	
	n	%	n	%	N	%	n	%
dry eyes								
No	104	100	47	47.00	28	100	179	77.16
Yes	0	0	53	53.00	0	0	53	22.84
Chi2 = 57.3; DF = 2; *p* < 0.001								
burning sensation in the eye								
No	104	100	37	37.00	28	100	169	72.84
Yes	0	0	63	63.00	0	0	63	27.16
Chi2 = 74.7; DF = 2; *p* < 0.001								
lachrymation								
No	103	99.04	32	32.00	28	100.00	163	70.26
Yes	1	0.96	68	68.00	0	0.00	69	29.74
Chi2 = 84.6; DF = 2; *p* < 0.001								
conjunctival redness								
No	104	100.00	48	48.00	28	100	180	77.59
Yes	0	0	52	52.00	0	0	52	22.41
Chi2 = 69.1; DF = 2; *p* < 0.001								
foreign body sensation								
No	104	100	48	48.00	28	100	180	77.59
Yes	0	0	52	52.00	0	0	52	22.41
Chi2 = 59.1; DF = 2; *p* < 0.001								
lash loss								
No	104	100	48	48.00	28	100	180	77.59
Yes	0	0	52	52.00	0	0	52	22.41
Chi2 = 11.3; DF = 2; *p* = 0.003								

**Table 3:** The comparison of right and left eye intraocular pressure (IOP) and visual acuity (VA) of uninfested (control group) and *Demodex folliculorum*-infested (*Demodex*-infested) patients (AM, arithmetic mean; SD, standard deviation; Med, median; Q1, lower quartile; Q2, upper quartile; and *p*, level of significance).

Parameter	Grup	AM	SD	Med.	Min	Max	Q1	Q2	*p*
IOP (mmHg)	Control	16.51	2.34	16.00	11.00	22.00	15.00	18.00	0.05
	*Demodex* infestation	15.82	3.09	15.00	9.00	27.00	14.00	17.00	
VA	Control	0.88	0.17	1.00	0.40	1.00	0.80	1.00	0.04
	*Demodex* infestation	0.79	0.23	0.90	0.10	1.00	0.60	1.00	

## Data Availability

The data presented in this study are available on request from the corresponding author.

## References

[1] Eberhardt M., Rammohan G. (2022). Blepharitis. StatPearls [Internet].

[2] Bernardes T.F., Bonfioli A.A. (2010). Blepharitis. Semin. Ophthalmol..

[3] Amescua G., Akpek E.K., Farid M., Garcia-Ferrer F.J., Lin A., Rhee M.K., Varu D.M., Musch D.C., Dunn S.P., Mah F.S. (2019). Blepharitis Preferred Practice Pattern®. Ophthalmology.

[4] Hosseini K., Bourque L.B., Hays R.D. (2018). Development and evaluation of a measure of patient-reported symptoms of blepharitis. Health Qual. Life Outcomes.

[5] Putnam C.M. (2016). Diagnosis and management of blepharitis: An optometrist’s perspective. Clin. Optom..

[6] Lindsley K., Matsumura S., Hatef E., Akpek E.K. (2012). Interventions for chronic blepharitis. Cochrane Database Syst. Rev..

[7] Liu X., Fu Y., Wang D., Huang S., He C., Yu X., Zhang Z., Kong D., Dai Q. (2022). Uneven index: A digital biomarker to prompt *Demodex* blepharitis based on deep learning. Front. Physiol..

[8] Desch C., Nutting M.B. (1972). *Demodex folliculorum* (Simon) and *D. brevis* Akbulatova of man: Redescription and reevaluation. J. Parasitol..

[9] Lacey N., Kavanagh K., Tseng S.C. (2009). Under the lash: *Demodex* mites in human diseases. Biochemist.

[10] Liu J., Sheha H., Tseng S.C. (2010). Pathogenic role of Demodex mites in blepharitis. Curr. Opin. Allergy Clin. Immunol..

[11] Czepita D., Kuźna-Grygiel W., Czepita M., Grobelny A. (2007). *Demodex folliculorum* and *Demodex brevis* as a cause of chronic marginal blepharitis. Ann. Acad. Med. Stetin..

[12] Jańczak D., Ruszczak A., Kaszak I., Gołąb E., Barszcz K. (2017). Clinical aspects of demodicosis in veterinary and human medicine. Med. Weter..

[13] Cheng A.M., Sheha H., Tseng S.C. (2015). Recent advances on ocular *Demodex* infestation. Curr. Opin. Ophthalmol..

[14] Bikowski J.B., Del Rosso J.Q. (2009). *Demodex* dermatitis: A retrospective analysis of clinical diagnosis and successful treatment with topical crotamiton. J. Clin. Aesthet. Dermatol..

[15] Bonnar E., Eustace P., Powell F.C. (1993). The Demodex mite population in rosacea. J. Am. Acad. Dermatol..

[16] Biernat M.M., Rusiecka-Ziółkowska J., Piątkowska E., Helemejko I., Biernat P., Gościniak G. (2018). Occurrence of *Demodex* species in patients with blepharitis and in healthy individuals: A 10-year observational study. Jpn. J. Ophthalmol..

[17] Arici C., Mergen B., Bahar Tokman H., Yildiz Tas A., Tokuc E., Ozturk Bakar Y., Sahin A. (2022). Investigation of the *Demodex* lid infestation with in vivo confocal microscopy versus light microscopy in patients with seborrheic blepharitis. Ocul. Immunol. Inflamm..

[18] Gonzalez-Salinas R., Karpecki P., Yeu E., Holdbrook M., Baba S.N., Ceballos J.C., Massaro-Corredor M., Corredor-Ortega C., Ramos-Betancourt N., Quiroz-Mercado H. (2022). Safety and efficacy of lotilaner ophthalmic solution, 0.25% for the treatment of blepharitis due to *Demodex* infestation: A randomized, controlled, double-masked clinical trial. Contact Lens Anterior Eye.

[19] Cheng A.M., Hwang J., Dermer H., Galor A. (2021). Prevalence of ocular demodicosis in an older population and its association with symptoms and signs of dry eye. Cornea.

[20] Shah P.P., Stein R.L., Perry H.D. (2022). Update on the management of *Demodex* blepharitis. Cornea.

[21] Zhu M., Cheng C., Yi H., Lin L., Wu K. (2018). Quantitative analysis of the bacteria in blepharitis with *Demodex* infestation. Front. Microbiol..

[22] Clifford C.W., Fulk G.W. (1990). Association of diabetes, lash loss, and *Staphylococcus aureus* with infestation of eyelids by *Demodex folliculorum* (Acari: Demodicidae). J. Med. Entomol..

[23] Lacey N., Ní Raghallaigh S., Powell F.C. (2011). *Demodex* mites—Commensals, parasites or mutualistic organisms?. Dermatology.

[24] Nicholls S.G., Oakley C.L., Tan A., Vote B.J. (2017). *Demodex* species in human ocular disease: New clinicopathological aspects. Int. Ophthalmol..

[25] Wolf R., Ophir J., Avigad J., Lengy J., Krakowski A. (1988). The hair follicle mites (*Demodex* spp.). Could they be vectors of pathogenic microorganisms?. Acta Derm. Venereol..

[26] O’Reilly N., Menezes N., Kavanagh K. (2012). Positive correlation between serum immunoreactivity to *Demodex*-associated bacillus proteins and erythematotelangiectatic rosacea. Br. J. Dermatol..

[27] Chudzicka-Strugała I., Gołębiewska I., Brudecki G., Elamin W., Zwoździak B. (2023). Demodicosis in different age groups and alternative treatment options-a review. J. Clin. Med..

[28] Perkins R.E., Kundsin R.B., Pratt M.V., Abrahamsen I., Leibowitz H.M. (1975). Bacteriology of normal and infected conjunctiva. J. Clin. Microbiol..

[29] Singer T.R., Isenberg S.J., Apt L. (1988). Conjunctival anaerobic and aerobic bacteria flora in paedatric versus adult subjects. Br. J. Ophthalmol..

[30] Kański J.J., Kubicka-Trząska A. (2004). The human physiological flora. Infectious Eye Diseases.

[31] Rhee M.K., Yeu E., Barnett M., Rapuano C.J., Dhaliwal D.K., Nichols K.K., Karpecki P., Mah F.S., Chan A., Mun J. (2023). *Demodex* blepharitis: A comprehensive review of the disease, current management, and emerging therapies. Eye Contact Lens.

[32] Yan Y., Yao Q., Lu Y., Shao C., Sun H., Li Y., Fu Y. (2020). Association between *Demodex* infestation and ocular surface microbiota in patients with *Demodex* blepharitis. Front. Med..

[33] Lee S.H., Oh D.H., Jung J.Y., Kim J.C., Jeon C.O. (2012). Comparative ocular microbial communities in humans with and without blepharitis. Investig. Ophthalmol. Vis. Sci..

[34] Spickett S.G. (1961). Studies on *Demodex folliculorum* Simon. Parasitology.

[35] Türk M., Oztürk I., Sener A.G., Küçükbay S., Afşar I., Maden A. (2007). Comparison of incidence of *Demodex folliculorum* on the eyelash follicule in normal people and blepharitis patients. Turkiye Parazitol. Derg..

[36] Lee S.H., Chun Y.S., Kim J.H., Kim E.S., Kim J.C. (2010). The relationship between *Demodex* and ocular discomfort. Investig. Ophthalmol. Vis. Sci..

[37] Bezza Benkaouha I., Le Brun C., Pisella P.J., Chandenier J., Lanotte P. (2015). Bacterial flora in blepharitis. J. Fr. Ophtalmol..

[38] Lacey N., Delaney S., Kavanagh K., Powell F.C. (2007). Mite-related bacterial antigens stimulate inflammatory cells in rosacea. Br. J. Dermatol..

[39] Li J., O’Reilly N., Sheha H., Katz R., Raju V.K., Kavanagh K., Tseng S.C. (2010). Correlation between ocular *Demodex* infestation and serum immunoreactivity to *Bacillus* proteins in patients with facial rosacea. Ophthalmology.

[40] O’Reilly N., Gallagher C., Reddy Katikireddy K., Clynes M., O’Sullivan F., Kavanagh K. (2012). *Demodex*-associated *Bacillus* proteins induce an aberrant wound healing response in a corneal epithelial cell line: Possible implications for corneal ulcer formation in ocular rosacea. Investig. Ophthalmol. Vis. Sci..

[41] Szkaradkiewicz A., Chudzicka-Strugała I., Karpiński T.M., Goślińska-Pawłowska O., Tułecka T., Chudzicki W., Szkaradkiewicz A.K., Zaba R. (2012). *Bacillus oleronius* and *Demodex* mite infestation in patients with chronic blepharitis. Clin. Microbiol. Infect..

[42] AlonsoDeVelasco E., Verheul A.F., Verhoef J., Snippe H. (1995). *Streptococcus pneumoniae*: Virulence factors, pathogenesis and vaccines. Microbiol. Rev..

[43] (1997). Prevention of pneumococcal disease recommendations of the advisory committee on immunization practices (ACIP). MMWR Morb. Mortal. Wkly. Rep..

[44] Rynerson J.M., Perry H.D. (2016). DEBS—A unification theory for dry eye and blepharitis. Clin. Ophthalmol..

